# Modulation of Wnt/β‐Catenin Pathway by *Aesculus hippocastanum* Extract Enhances Temozolomide Sensitivity in Glioblastoma Cells

**DOI:** 10.1111/jcmm.70979

**Published:** 2026-02-16

**Authors:** Sarah Adriana Scuderi, Alessio Ardizzone, Deborah Mannino, Nicoletta Palermo, Fabiola De Luca, Antonio Catalfamo, Michela Campolo, Emanuela Esposito, Irene Paterniti

**Affiliations:** ^1^ Genetics and Pharmacogenetics Unit “Gaetano Martino” University Hospital Messina Italy; ^2^ UniCamillus‐Saint Camillus International University of Health Sciences Rome Italy; ^3^ Research Operative Unit of Neuropharmacology and Translational Neurosciences Oasi Research Institute, IRCCS Italy; ^4^ Department of Biomedical and Dental Sciences and Morphofunctional Imaging University of Messina Messina Italy; ^5^ Department of Chemical, Biological, Pharmaceutical and Environmental Sciences University of Messina Messina Italy

**Keywords:** *Aesculus hippocastanum*, chemotherapy, glioblastoma (GB), horse chestnut extract (HCE), temozolomide (TMZ), Wnt/β‐catenin signalling pathway

## Abstract

Glioblastoma (GB) is a highly aggressive brain tumour with a poor prognosis and limited responsiveness to standard chemotherapy, particularly temozolomide (TMZ), due to intrinsic resistance mechanisms. This study investigates the potential of *Aesculus hippocastanum,* known as horse chestnut extract (HCE), to enhance the therapeutic efficacy of TMZ in GB cells through modulation of the Wnt/β‐catenin signalling pathway. Combined treatment of HCE (500 μg/mL) and TMZ (100 μM) significantly reduced cell viability and inhibited wound healing and colony formation compared to either agent alone at 48 h. Notably, the expression of β‐catenin and Wnt‐1 was significantly reduced in the combination group, followed by a significant downregulation of Nestin and β3‐tubulin, markers of glioma stem‐like cells and aggressiveness, respectively. Furthermore, apoptotic activity was significantly increased following the combined treatment. In a 3D U87‐spheroid model, the combination therapy resulted in a substantial reduction in spheroid area, suggesting impaired tumour growth. Propidium iodide (PI) staining revealed increased membrane permeability in cells treated with the combination, which was accompanied by an increase in p53 expression, supporting the induction of apoptosis. Collectively, these findings demonstrate that HCE increases the cytotoxic effects of TMZ by inhibiting Wnt/β‐catenin signalling, reducing tumour stemness, and promoting apoptotic pathways in GB cells.

## 1 Introduction

Glioblastoma (GB) is the most common and aggressive primary malignant tumour of the central nervous system (CNS) in adults, classified as grade IV astrocytoma by the World Health Organization (WHO) [1]. It is characterised by rapid growth, diffuse infiltration into surrounding brain tissue, extensive heterogeneity, and a pronounced tendency for recurrence [2]. These features, combined with a highly immunosuppressive tumour microenvironment (TME) and a dynamic, therapy‐adaptable phenotype, contribute to its poor prognosis [3].

Despite significant advances in neurosurgical techniques, radiotherapy, and chemotherapeutic protocols, the overall therapeutic success remains limited [4]. The current standard of care involves maximal safe surgical resection followed by concurrent radiotherapy and adjuvant chemotherapy with temozolomide (TMZ), an oral alkylating agent [5]. However, this regimen offers only a modest extension of survival, with a median overall survival of approximately 14 months and a 5‐year survival rate of 5% [6, 7]. Tumour recurrence is almost universal, and recurrent GB often exhibits even greater resistance to conventional treatments [8]. Indeed, a major obstacle to successful therapy is the intrinsic and acquired resistance of GB cells to chemotherapy, particularly to TMZ [8]. This resistance is multifactorial, involving both genetic and epigenetic mechanisms. One of the key determinants is the expression of O6‐methylguanine‐DNA methyltransferase (MGMT), a DNA repair enzyme that reverses the cytotoxic lesions induced by TMZ [9]. Methylation of the MGMT promoter, which leads to reduced enzyme expression, is associated with improved TMZ response and better clinical outcomes; however, even in patients with favourable MGMT methylation status, resistance often develops over time [10]. Beyond MGMT, other resistance mechanisms include the presence of a subpopulation of glioma stem‐like cells (GSCs) with enhanced DNA repair capacity [11], upregulation of drug efflux pumps (such as ABC transporters) [12], alterations in apoptotic signalling pathways [13], metabolic rewiring [14], and adaptive activation of pro‐survival pathways such as PI3K/Akt, MAPK, and Wnt/β‐catenin [15]. These mechanisms allow tumour cells not only to survive chemotherapeutic stress but also to evolve rapidly under therapeutic pressure, leading to treatment failure and disease progression.

In particular, growing evidence proposes the modulation of the Wnt/β‐catenin signalling pathway as a promising approach to counteract GB chemoresistance [16, 17]. This pathway regulates key cellular processes such as proliferation, survival, migration, and the maintenance of GSCs, which are highly resistant to conventional therapies [18]. Aberrant activation of Wnt/β‐catenin in GB promotes the transcription of oncogenic targets like Cyclin D1, MYC, and Bcl‐2, supporting tumour growth and evasion of apoptosis [16]. Inhibition of this pathway has been shown to sensitise glioma cells to TMZ by enhancing TMZ‐induced cytotoxicity [19]. Therefore, targeting Wnt/β‐catenin may provide a synergistic mechanism to potentiate the efficacy of standard chemotherapeutic regimens.

However, the anatomical location of GB within the CNS poses unique therapeutic challenges, including limited drug penetration across the blood–brain barrier (BBB), which restricts the bioavailability of many potentially effective agents [20]. This pharmacological limitation further complicates the development of novel therapeutics, underscoring the urgent need for more effective, targeted, and BBB‐permeable strategies.

Given these clinical and molecular complexities, there is a growing interest in exploring combinatorial and adjuvant therapies that can sensitise GB cells to TMZ and other treatments. This includes the investigation of small‐molecule inhibitors, immunotherapies, and natural compounds capable of modulating key pathways involved in GB [21, 22, 23].

Natural compounds derived from medicinal plants are gaining traction as complementary therapeutic agents; these species often exhibit multi‐target activities and can modulate key signalling cascades involved in cancer progression [24]. Among these, *Aesculus hippocastanum* , commonly known as horse chestnut extract (HCE), has been traditionally used for its anti‐inflammatory, antioxidant, and vasoprotective properties [25]. Its main bioactive component, escin, has demonstrated antitumor effects in several cancer models by inhibiting cell proliferation, inducing apoptosis, and modulating inflammatory pathways [26, 27, 28].

For instance, in breast cancer (BC), escin reduced tumour growth in vivo and in vitro by inhibiting the expression of GPX4 and contributing to ferroptosis [27]. Moreover, it has also been reported to increase the cytotoxic effect of cisplatin by modulating the apoptosis pathway [27]. In pancreatic cancer, escin decreased cell survival and induced apoptosis via downregulation of NF‐κB‐related proteins and cyclin D, sensitising pancreatic cancer cells to chemotherapy as well [28]. Likewise, in colorectal cancer, escin inhibited cell viability, colony formation, and induced DNA damage through p‐ATM and γH2AX upregulation [29]. Taken together, these data indicate that escin can modulate convergent cancer‐relevant pathways across different malignancies and interact with standard chemotherapeutics, providing a rationale for further investigation in additional cancer types. The potential of HCE to influence Wnt/β‐catenin signalling in GB cells, as far as we know, has not been fully explored. Moreover, its capacity to enhance the efficacy of standard chemotherapeutic agents such as TMZ remains to be determined. Thus, given this background, we hypothesised that HCE might modulate Wnt/β‐catenin activity in GB cells and, in doing so, sensitise them to TMZ‐induced cytotoxicity. Thus, in the present study, we investigated the effects of HCE in combination with TMZ on GB viability, proliferation, and apoptosis, with a particular focus on the Wnt/β‐catenin pathway as a potential mechanistic link.

## 2 Materials and Methods

### 2.1 Materials

#### 2.1.1 Materials

HCE (*Aesculus hippocastanum L*.) water‐soluble extract obtained from seeds was provided by the Chemical Department of the University of Messina, and it was offered in the form of a dry powder.

HCE extract was prepared as previously described [30]. The qualitative and quantitative analysis of HCE was conducted using an HPLC‐DAD‐MS system [30]. Chromatographic separation was performed using an Agilent Series HPLC system, coupled with a diode array detector and an ion trap mass spectrometer. Separation was achieved on a Poroshell 120 EC‐C18 column (4.6 × 100 mm, 2.7‐Micron; Agilent Technologies, Santa Clara, CA, USA), using a binary solvent system composed of methanol with 1 mM HCOONH_4_ and 1% HCOOH (solvent A) and H_2_O with 1 mM HCOONH_4_ and 1% HCOOH (solvent B). The elution gradient: 0 min 70% A + 30% B; 7 min 100% A; 13 min 100% A; 13.1 min 70% A + 30% B; 20 min 70% A + 30% B. Flow: 0.3 mL/min, temperature: 30°C. Constituents' identification was performed through MSⁿ fragmentation patterns and confirmed by direct comparison with a certified β‐escin reference standard. Quantification was based on a calibration curve constructed from six different concentrations of the reference compound (US Pharmacopeial Convention, 99% purity, batch No. R023G0) [30]. The detailed phytochemical profile of HCE, including the content of escin, is provided in Table S1. The standardised dry extract used in this study contains 19.55% escin. Unless otherwise specified, all other reagents and materials used in the experimental procedures were obtained from Sigma‐Aldrich.

### 2.2 Cell Line and Culture Conditions

Human U87 MG (ATCC HTB‐14 *Homo sapiens* brain likely glioblastomas) and A172 (ATCC CRL‐1620 *Homo sapiens* brain likely glioblastomas) cell lines were obtained from ATCC (American Type Culture Collection, Rockville, MD, USA). U87 and A172 cells were cultured in 75 cm^2^ flasks in Dulbecco's modified Eagle's medium (DMEM—Sigma‐Aldrich Catalogue No. D5030; St. Louis, MO, USA) supplemented with 10% fetal bovine serum (FBS, Sigma‐Aldrich Catalogue No. 12103C St. Louis, MO, USA), L‐glutamine (GlutaMAX, ThermoFisher Scientific Catalogue No. 35050061; Waltham, MA, USA), and antibiotics (Penicillin 1000 units—Streptomycin 0.1 mg/L, Sigma‐Aldrich Catalogue No. P4333; St. Louis, MO, USA). Cells were maintained in the incubator with a humidified atmosphere containing 5% CO_2_ at 37°C.

### 2.3 Cell Treatments

U87 and A172 cells were treated with increasing concentrations of HCE (from 62.5–800 μg/mL) and TMZ (100 μM) for 24 h and 48 h, alone or in combination. A stock solution of HCE (1 mg/mL) was prepared in basal medium. TMZ (Cat. No. T2577, Sigma‐Aldrich, Milan, Italy) was first solubilised in dimethyl sulfoxide (DMSO) and then dissolved in basal medium to obtain a final concentration of 100 μM. The concentrations of HCE and TMZ were chosen according to previous in vitro studies [31, 32].

### 2.4 Cell Viability Assay

Cell viability was measured using a mitochondria‐dependent dye for live cells (tetrazolium dye; MTT), as previously described [33]. U87 and A172 cells were seeded in a 96‐well plate at about 10,000 cells per well. After 24 h, cells were treated with increasing concentrations of HCE (from 62.5 to 800 μg/mL) and TMZ (100 μM), alone or in combination, for 24 and 48 h to determine higher concentrations with high toxicity. After, U87 and A172 cells were incubated at 37°C with MTT (0.2 mg/mL) for 1 h. Absorbance was read at 570 nm using a microplate reader.

### 2.5 Wound Healing Assay

U87 and A172 cells were plated on a microscope slide (2 × 10^5^) in a final volume of 1 mL. After 24 h, medium was removed, and cells were washed with phosphate‐buffered saline (PBS) to remove any debris. A linear scratch/wound was made using a sterile 200 μL pipette tip [33]. After that, HCE (500 μg/mL) and TMZ (100 μM) were added to the cells for 48 h. Later, medium was removed, and cells were fixed with paraformaldehyde (PFA) 4% for 30 min and then stained with crystal violet solution (1:1). The images were captured using the microscope (Nikon Eclipse Ci‐L, NIKON CORPORATION, Tokyo, Japan) at 2× magnification. Wound area was recorded at 48 h by using Image J software.

### 2.6 Colony Formation Assay

1000 cells/well were seeded in 6‐well plates. After 24 h, U87 and A172 cells were treated with HCE (500 μg/mL) and TMZ (100 μM) for 48 h, alone or in combination. After 48 h of treatment, media supplemented with 10% FBS was added to the cells. After 10 days, cells were washed with PBS, fixed in PFA for 30 min, and stained with crystal violet solution (1:1) [34]. The stained cells were photographed using the microscope (Nikon Eclipse Ci‐L, NIKON CORPORATION, Tokyo, Japan) at 10× magnification. Subsequently, colony quantification was conducted.

### 2.7 Immunoblotting

Western blot was performed in U87 (monolayer and spheroids) and A172 cells as previously described [35]. The following primary antibodies were used: β‐catenin (1:500, Santa Cruz Biotechnology, Dallas, TX, USA; sc‐133,238), Wnt‐1 (1:500, Invitrogen, Waltham, USA, Cat. No. 36‐5800), Bcl‐2 (1:500, Santa Cruz Biotechnology, Dallas, TX, USA; sc‐7382), p53 (1:500, Santa Cruz Biotechnology, Dallas, TX, USA; sc‐126), and Bax (1:1000, Invitrogen, Cat. No. PA5‐11378). Glyceraldehyde‐3‐Phosphate Dehydrogenase (GAPDH) (1:1000; Santa Cruz Biotechnology; Dallas, TX, USA, sc‐365062 HRP) was used as the loading control. Image J software was used to quantify the protein expression.

### 2.8 NOx Assay

Nitrite levels were measured in U87 cells' supernatant using the Griess method as described before described [36].

### 2.9 MitoTracker Red

U87 cells were seeded on a microscopic slide (2 × 10^5^) in a final volume of 1 mL. After 48 h of treatment with HCE (500 μg/mL) and TMZ (100 μM), alone or in combination, MitoTracker Red CMXRos (Thermo Fisher Scientific, Cat. No. M7512) (500 nM) was added to the cells for 45 min according to the manufacturer's instructions. Afterward, U87 cells were washed with PBS and fixed with 4% PFA for 30 min. Images were captured at 40× magnification using a fluorescence microscope (Nikon Eclipse Ci‐L, NIKON CORPORATION, Tokyo, Japan).

### 2.10 TUNEL Assay

TUNEL (terminal deoxynucleotidyl transferase) In Situ Cell Death Detection Kit, TMR red (Roche Cat. No. 12156792910) was used to evaluate cell death according to the manufacturer's instructions.

Images were captured at 40× magnification using a fluorescence microscope (Nikon Eclipse Ci‐L, NIKON CORPORATION, Tokyo, Japan).

### 2.11 ELISA Assay

The levels of malondialdehyde (MDA) (Human MDA ELISA Kit, Assay Genie; Cat. No. UNFI0048), Reactive Oxygen Species Modulator 1 (ROMO‐1) (Human ROMO‐1 ELISA kit, Aviva Systems Biology, Cat. No. OKEH01371), Glutathione (GSH) (GSH ELISA kit, Cloud‐Clone Corp, Cat. No. CEA294Ge), and 3‐Nitrotyrosine (3‐NT) (Human 3‐NT ELISA Kit Elabscience; Cat. No. E‐EL‐0040) were measured in U87 cell supernatants according to the manufacturer's instructions. The levels of phospho‐GSK‐3β (Ser9) and total GSK‐3β (Human ELISA Kit, RayBiotech; Cat. No. PEL‐GSK3b‐S9‐T) were evaluated in U87 cell lysates. The absorbance was measured at 450 nm using the microplate reader GloMax Discover (Promega).

### 2.12 Measurement of Intracellular ROS

Reactive Oxygen Species (ROS) Fluorometric Assay Kit (Assay Genie; Cat. No. MAES0112) was used to measure intracellular ROS levels according to the manufacturer's instructions. Briefly, after seeding U87 cells in 12 well‐plates (3 × 10^4^ cells/well), cells were treated with HCE (500 μg/mL) and TMZ (100 μM) alone or in combination for 48 h and incubated at 37°C and 5% CO_2_. After 48 h, 2,7‐dichlorofluorescin diacetate (DCFH‐DA) (15 μM) was added to the cells and incubated for 1 h at 37°C. Cells were collected and resuspended in PBS for fluorescence detection (Ex/Em = 500 nm/525 nm) using the fluorescence microplate reader GloMax Discover (Promega).

### 2.13 3D Spheroid Cell Culture, Treatments and Area Measurement

U87 cells (10,000 cells/well) were seeded in 96‐well round‐bottom plates pre‐coated with 2% agarose, in a final volume of 100 μL per well. Plates were incubated at 37°C in a humidified atmosphere with 5% CO_2_. Spheroid formation was monitored after 24 h. On day 3 post‐seeding, U87 spheroids were treated with HCE (500 μg/mL) and TMZ (100 μM), either individually or in combination, for a period of 4 days. Spheroid area was assessed by Image J at days 1, 3, and 7 until the end of the experiment. Images were captured at 4× magnification. Independent spheroid batches were generated for each specific technique.

### 2.14 Haematoxylin and Eosin (H&E) Staining

After treatment, U87 spheroids were fixed in PFA 4%, embedded into paraffin blocks, and cut at 4 μm as described by Yoshimoto et al. [37]. Spheroids were then stained with haematoxylin and eosin (H&E) to assess tissue morphology. Images were captured at 10× magnification (Nikon Eclipse Ci‐L microscope, NIKON CORPORATION, Tokyo, Japan). Independent spheroid batches were generated for each specific technique.

### 2.15 Propidium Iodide (PI) Staining

PI staining (Cat. No. P4864, Sigma Aldrich) was performed to evaluate cell membrane integrity and detect dead or late‐apoptotic cells in U87 spheroids [38]. PI was added at a concentration of 5 μg/mL. Images were captured at 2× magnification using a fluorescence microscope (Nikon Eclipse Ci‐L, NIKON CORPORATION, Tokyo, Japan). Integrated density was measured by using Image J. Independent spheroid batches were generated for each specific technique.

### 2.16 Immunofluorescence

Immunofluorescence staining was executed on U87 monolayer and U87 spheroids as previously described [39, 40]. The following primary antibodies were used: Nestin (1:100, Arigo Laboratories, Cat. No. ARG52345), β‐3 tubulin (1:100, Santa Cruz Biotechnology, Dallas, TX, USA; sc‐80005), Bax (1:100, Invitrogen; Cat. No. MA5‐14003), and p53 (1:100, Santa Cruz Biotechnology, Dallas, TX, USA; sc‐126). Later, incubation with the secondary antibody Alexa Fluor‐488 or Alexa Fluor‐594 (1:1000 in PBS, *v*/*v*, Invitrogen; Waltham, MA, USA) was performed for 3 h. Following incubation, sections were washed in PBS and 4′,6′‐diamidino‐2‐phenylindole (DAPI; Hoechst, Frankfurt, Germany) (2 μg/mL) was added to the cells for nuclear staining. Images were captured at 10×, 20×, and 40× magnifications using a fluorescence microscope (Nikon Eclipse Ci‐L, NIKON CORPORATION, Tokyo, Japan). Independent spheroid batches were generated for each specific technique.

### 2.17 Statistical Analysis

Data were obtained from 3 independent experiments; then, the results were analysed using the software GraphPad Prism 9.5.1 and expressed as means ± standard error of the mean (SEM). Following the normality tests, statistical analysis was performed using a One‐Way or Two‐Way analysis of variance (ANOVA) followed by Bonferroni correction test. Only values at *p* < 0.05 were considered statistically significant.

## 3 Results

### 3.1 Effects of HCE and TMZ Combination on GB Cell Viability

To evaluate the impact of HCE on GB cell viability and its potential to enhance TMZ sensitivity, U87 and A172 cells were treated with increasing concentrations of HCE (62.5, 125, 250, 500, and 800 μg/mL), either alone or in combination with TMZ (100 μM) for 24 and 48 h.

**FIGURE 1:**
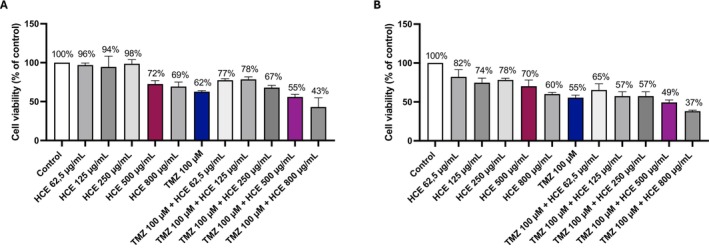
Evaluation of HCE cytotoxicity alone or in combination with TMZ in U87 cells. Cell viability was assessed by MTT assay after 24 and 48 h treatment with increasing concentrations of HCE (62.5, 125, 250, 500, and 800 μg/mL), alone or in combination with TMZ (100 μM) (A, B). Data are representative of three independent experiments.

HCE alone induced a reduction in U87 and A172 cell viability at both time points at higher concentrations, with a more pronounced cytotoxic effect observed at 48 h (Figure 1A,B; Figure S1A,B). TMZ monotherapy (100 μM) produced a considerable cytotoxic effect as well (Figure 1A,B; Figure S1A,B). However, co‐treatment with HCE led to a marked enhancement of TMZ‐induced cytotoxicity, particularly at higher HCE concentrations. The combination of TMZ with HCE at 250 μg/mL, 500 μg/mL, and 800 μg/mL significantly reduced GB cell viability compared to either agent alone at 48 h, suggesting a synergistic interaction. Notably, the combination of TMZ with HCE 800 μg/mL induced excessive cytotoxicity, rendering subsequent mechanistic analyses unfeasible due to likely poor cell viability. Therefore, for all further experiments, we selected the 500 μg/mL concentration of HCE for combination treatments, as it provided strong cytotoxic synergy with TMZ at 48 h in GB cells, while maintaining acceptable cell viability for downstream assays.

### 3.2 Effects of HCE and TMZ Combination on Colony Formation and Wound Closure in GB Cells

To further assess the anti‐proliferative and anti‐migratory effects of the HCE and TMZ combination, we performed wound healing and colony formation assays in U87 and A172 cells.

**FIGURE 2:**
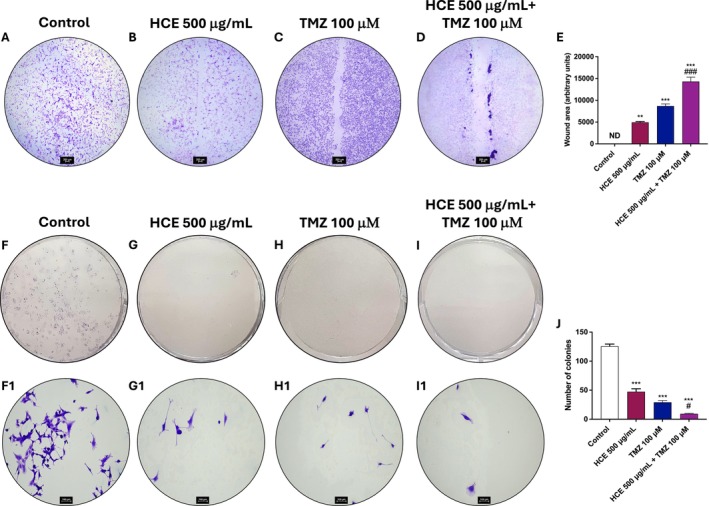
HCE and TMZ combination impairs migration and clonogenic ability of U87 cells. Wound healing (A–E) and colony formation assays (F–I, F1‐I1) were performed on U87 cells treated with HCE (500 μg/mL) and TMZ (100 μM), alone or in combination, for 48 h. Data are representative of three independent experiments. One‐Way ANOVA. (E) **p < 0.01 versus Control; ***p < 0.001 versus Control; ###*p* < 0.001 versus TMZ. ND = not detected. (J) ***p < 0.001 versus Control; #*p* < 0.05 versus TMZ.

In the wound healing assay, monotherapy with either HCE or TMZ partially inhibited wound closure at 48 h (Figure 2B,C; Figure S2B,C), indicating a reduction in cell migration compared to the control group (Figure 2A; Figure S2A). Nevertheless, the combination treatment resulted in a significant impairment of wound closure compared to either treatment alone (Figure 2D, score 2E; Figure S2D, score S2E), suggesting that the co‐administration of HCE and TMZ markedly impairs GB cells' migration.

Similarly, in the colony formation assay, both HCE and TMZ monotherapies led to a reduction in the number of colonies (Figure 2G–H1; Figure S2G–H1) compared to the control group (Figure 2F,F1; Figure S2F,F1), indicating a reduction of clonogenic potential. However, the combination of HCE and TMZ produced a significantly more pronounced effect, with a marked decrease in colony number and almost complete inhibition of colony outgrowth (Figure 2I,I1, score 2J; Figure S2I,I1, score S2J).

### 3.3 Effects of HCE and TMZ Combination on Wnt/β‐Catenin Signalling Pathway

To investigate whether the observed chemosensitizing effects of HCE were mediated through modulation of the Wnt/β‐catenin signalling pathway, U87 cells were treated with HCE (500 μg/mL), TMZ (100 μM), or their combination for 48 h.

**FIGURE 3:**
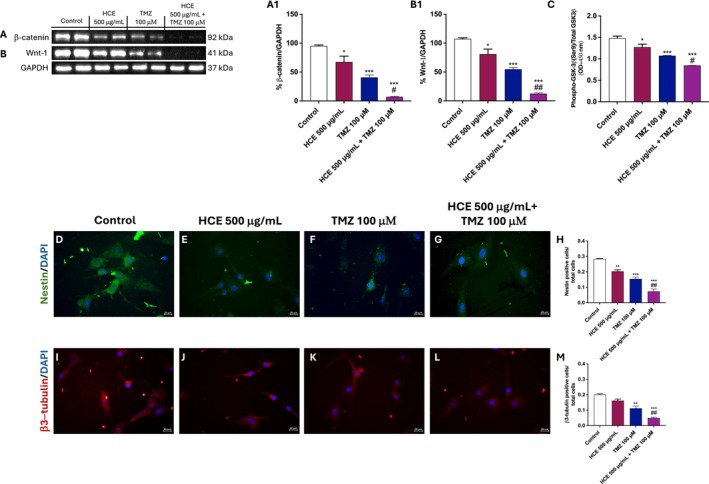
HCE and TMZ combination inhibits Wnt/β‐catenin signalling and modulates glioma stemness and differentiation markers in U87 cells. U87 cells were treated with HCE (500 μg/mL), TMZ (100 μM), or their combination, for 48 h. Western blot analysis of β‐catenin and Wnt‐1 was performed (A, B; A1, B1). ELISA kit for phospho‐GSK‐3β (Ser9) and total GSK‐3β was shown in (C). Nestin (D–H) and β3‐tubulin (I–M) were assessed by IF. Images of IF were captured at 40× magnification. Data are representative of three independent experiments. One‐Way ANOVA. (A1) *p < 0.05 versus Control; ***p < 0.001 versus Control; #*p* < 0.05 versus TMZ. (B1) *p < 0.05 versus Control; ***p < 0.001 versus Control; ##*p* < 0.01 versus TMZ. (C) *p < 0.05 versus Control; ***p < 0.001 versus Control; #*p* < 0.05 versus TMZ. (H) **p < 0.01 versus Control; ***p < 0.001 versus Control; ##*p* < 0.01 versus TMZ. (M) **p < 0.01 versus Control; ***p < 0.001 versus Control; ##*p* < 0.01 versus TMZ.

Treatment with HCE alone resulted in a moderate downregulation of β‐catenin and Wnt‐1 expression compared to control, suggesting an inhibitory effect (Figure 3A,B respectively, densitometric analysis A1 and B1, respectively). TMZ alone also considerably reduced β‐catenin and Wnt‐1 expression (Figure 3A,B respectively, densitometric analysis A1 and B1 respectively). However, the combination treatment markedly reduced their expression, indicating effective inhibition of β‐catenin/Wnt pathway activation (Figure 3A,B respectively, densitometric analysis A1 and B1 respectively). Moreover, we evaluated the levels of phospho‐GSK‐3β (Ser9) and total GSK‐3β in U87 cell lysates by ELISA kit, showing that the combinatory therapy significantly reduced their levels compared to HCE and TMZ treatments alone (Figure 3C).

To further explore the modulation of the Wnt/β‐catenin pathway, we assessed the expression of Nestin, a marker of glioma stem‐like cells, and β3‐tubulin, a neuronal differentiation marker. Treatment with HCE alone significantly reduced the number of Nestin positive cells but not β3‐tubulin (Figure 3E,J) compared to the control group (Figure 3D,I). Similar but more consistent results were obtained by TMZ treatment alone, which successfully reduced the number of positive cells of both markers (Figure 3F,K). Interestingly, the combination treatment resulted in a more significant decrease in Nestin and β3‐tubulin (Figure 3G,L, score 3H,M).

### 3.4 Effects of HCE and TMZ Combination on Mitochondrial Damage and Apoptosis Pathway

**FIGURE 4:**
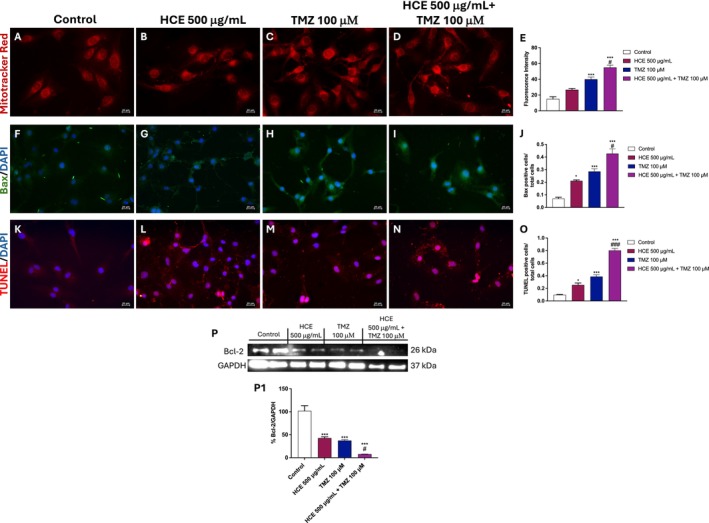
Combined HCE and TMZ treatment induces mitochondrial damage and activates apoptosis in U87 cells. Mitochondrial damage was assessed by Mitotracker Red staining (A–E). IF of Bax (F–J) and TUNEL assay (K–O) were performed. Images were captured at 40× magnification. Western blot analysis was performed to evaluate the expression of Bcl‐2 (P, P1). Data are representative of three independent experiments. One‐Way ANOVA. (E) ***p < 0.001 versus Control; #*p* < 0.05 versus TMZ. (J) *p < 0.05 versus Control; ***p < 0.001 versus Control; #*p* < 0.05 versus TMZ. (O) *p < 0.05 versus Control; ***p < 0.001 versus Control; ###*p* < 0.001 versus TMZ. (P) ***p < 0.001 versus Control; #*p* < 0.05 versus TMZ.

Considering the key role of apoptosis in GB, we decided to explore whether the combined treatment of HCE and TMZ induces mitochondrial dysfunction and activates apoptosis in U87 cells. Mitotracker Red staining revealed an altered mitochondrial morphology in U87 cells treated with TMZ alone, indicative of early mitochondrial stress, compared to control and HCE groups (Figure 4A–C, score 4E). These alterations were markedly more pronounced in the combination group (Figure 4D).

IF for Bax showed an increased number of positive cells in U87 cells exposed to the combination treatment compared to each treatment alone and the control group (Figure 4F–I, score 4J).

The pro‐apoptotic effect of HCE and TMZ was further confirmed by TUNEL staining, which showed a significant increase in TUNEL‐positive cells in the combined treatment group compared to either treatment alone or control, indicating enhanced activation of apoptosis (Figure 4K–N, score 4O).

In line with these observations, western blot analysis demonstrated a downregulation of the anti‐apoptotic protein Bcl‐2 in U87 cells treated with the combination therapy (Figure 4P,P1), confirming the previous results. To validate our data on the apoptosis pathway, we also investigated the expression of p53 and Bax in the A172 cell line (Figure S3).

### 3.5 Effects of HCE and TMZ Combination on Oxidative and Nitrosative Stress

**FIGURE 5:**
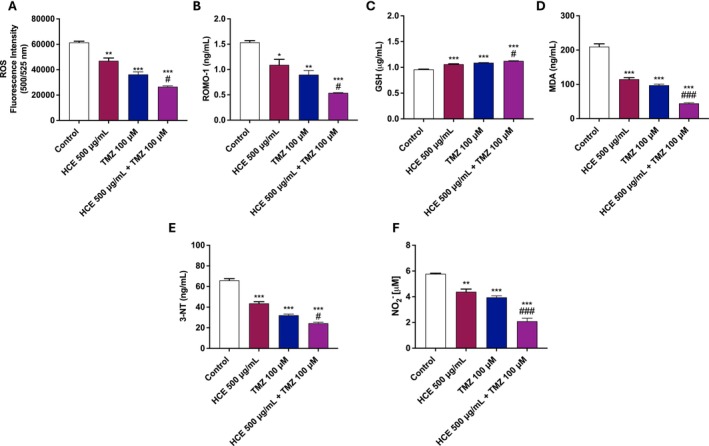
Effects of HCE and TMZ on oxidative and nitrosative stress in U87 cells. U87 cells were treated with HCE (500 μg/mL), TMZ (100 μM), or their combination for 48 h. Levels of ROS (A), ROMO‐1 (B), GSH (C), MDA (D), 3‐NT (E), and NO_2_^−^ (F) were assessed. Data are representative of three independent experiments. One‐Way ANOVA. (A) **p < 0.01 versus Control; ***p < 0.001 versus Control; #*p* < 0.05 versus TMZ. (B) *p < 0.05 versus Control; **p < 0.01 versus Control; ***p < 0.001 versus Control; #*p* < 0.05 versus TMZ. (C) ***p < 0.001 versus Control; #*p* < 0.05 versus TMZ. (D) ***p < 0.001 versus Control; ###*p* < 0.001 versus TMZ. (E) ***p < 0.001 versus Control; #*p* < 0.05 versus TMZ. (F) **p < 0.01 versus Control; ***p < 0.001 versus Control; ###*p* < 0.001 versus TMZ.

To assess the antioxidant effects of HCE and TMZ combination, several oxidative stress markers were analysed in U87 cells. Treatment with HCE (500 μg/mL) and TMZ (100 μM) alone led to a modest reduction in intracellular ROS levels, ROMO‐1, and MDA, compared to the control group (Figure 5A,B,D). Notably, co‐treatment with HCE and TMZ resulted in a more significant decrease in pro‐oxidative species compared to each treatment alone (Figure 5A,B,D).

In line with this, GSH levels were appreciably increased in the combination group compared to the control group and treatments alone (Figure 5C), further supporting a positive antioxidant effect.

Moreover, we decided to evaluate the effect of HCE and TMZ on nitrosative stress markers as 3‐NT and NO_2_^−^, demonstrating that the combined treatment significantly reduced their levels compared to the control group and each treatment alone (Figure 5E,F).

### 3.6 Effects of HCE and TMZ Combination on U87 Spheroid Area

**FIGURE 6:**
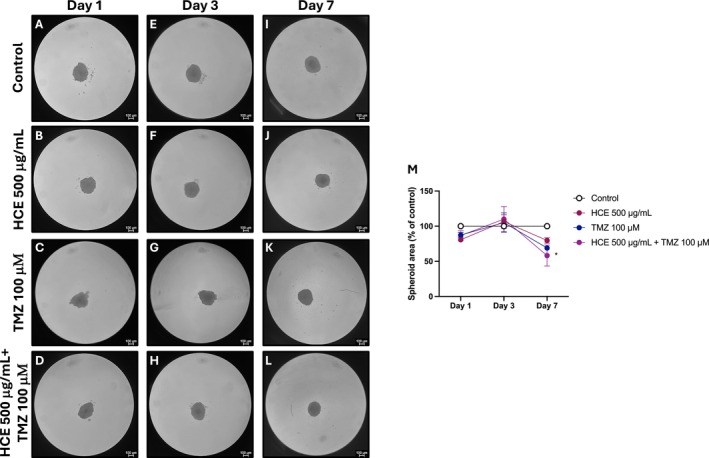
Effects of HCE and TMZ on U87 spheroid area over time. U87 spheroids were treated with HCE (500 μg/mL), TMZ (100 μM), or their combination, and spheroid area was assessed at Day 1 (A–D), Day 3 (E–H), and Day 7 (I–L, score M). Data are representative of three independent experiments. Two‐Way ANOVA. (M) *p < 0.05 versus Control.

To assess the impact of HCE and TMZ on 3D tumour growth, U87 spheroids were treated with HCE (500 μg/mL), TMZ (100 μM), or their combination for 4 days, and spheroid area was measured at Day 1, 3, and 7. No significant differences in spheroid area were observed among the experimental groups at Day 1 (Figure 6A–D) and Day 3 (Figure 6E–H). However, at Day 7, co‐treatment with HCE and TMZ resulted in a significant reduction in spheroid area compared to control and single‐agent treatments (Figure 6I–L, score 6 M).

### 3.7 Effects of HCE and TMZ Combination on Morphological Alterations and Cell Death in 3D Spheroid Model

To further investigate the effects of HCE and TMZ on 3D tumour architecture and cell viability, we performed H&E and PI staining.

**FIGURE 7:**
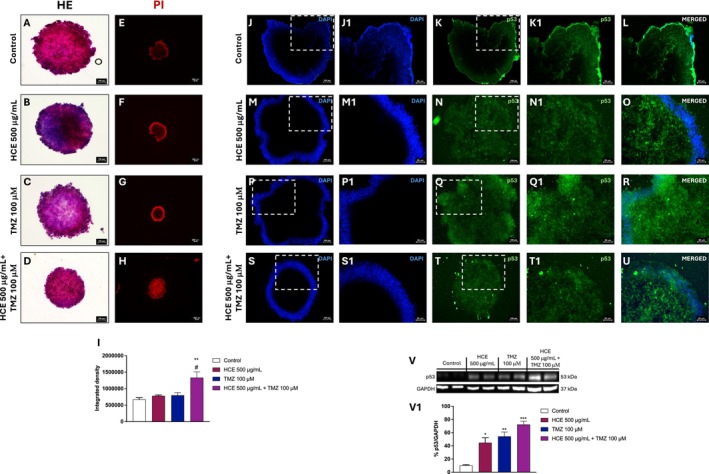
Effects of HCE and TMZ on morphology and cell death in U87 spheroids. U87 spheroids were treated with HCE (500 μg/mL), TMZ (100 μM), or their combination. H&E staining was executed (A–D). PI staining (E–I) and IF for p53 were performed (J–U). Western blot analysis for p53 was shown (V, V1). Images were captured at 2×, 10×, and 20× magnifications. One‐Way ANOVA. (I) **p < 0.01 versus Control; #*p* < 0.05 versus TMZ. (V1) *p < 0.05 versus Control; **p < 0.01 versus Control; ***p < 0.001 versus Control.

H&E staining revealed preserved spheroid architecture in control samples (Figure 7A) compared to treatment with HCE or TMZ alone (Figure 7B,C respectively). However, the combination treatment led to enlarged necrotic cores and reduced sizes (Figure 7D).

PI staining confirmed these findings, showing minimal fluorescence in control (Figure 7E) and monotherapy groups (Figure 7F,G, respectively), while the combination treatment induced a robust increase in PI‐positive nuclei (Figure 7H,I), indicating extensive loss of membrane integrity and late‐stage cell death within the spheroids.

Moreover, we decided to evaluate the effect of HCE and TMZ on p53, a marker of apoptosis, by IF. Compared to the control group (Figure 7J–L), IF analysis for p53 revealed a minimal increase in p53 positive cells in TMZ‐treated spheroids (7P‐P1, 7Q‐Q1, and 7R), and compared to the HCE group (Figure 7M–O); however, a pronounced upregulation was detected in the combination group (Figure 7S–U). Data about p53 was also assessed by western blot analysis (Figure 7V,V1), showing that the combination increased p53 expression compared to treatments alone but not significantly.

## 4 Discussion

GB is the most aggressive and lethal primary brain tumour in adults, characterised by high proliferative capacity, invasiveness, and resistance to standard therapies [35, 39]. Despite the available strategies as surgical resection followed by radiotherapy and chemotherapy, prognosis remains poor [39]. This highlights the urgent need for novel therapeutic approaches. Recently, increasing attention has been directed toward the use of natural compounds as adjuvant agents to conventional chemotherapy, aiming to enhance efficacy and overcome drug resistance [41, 42].

In this context, our study investigated the combined effects of HCE and TMZ on GB cells, using both monolayer cultures and 3D spheroid models to better mimic tumour behaviour in vitro.

In U87 and A172 monolayer models, the combined treatment of HCE (500 μg/mL) and TMZ (100 μM) led to a more pronounced reduction in cell viability compared to either agent alone, suggesting a synergistic cytotoxic effect, especially at 48 h. Moreover, the combination of HCE and TMZ markedly reduced the number of colony‐forming cells and inhibited wound closure in both GB cell lines. Several signalling pathways are often aberrantly activated in GB, including the Wnt/β‐catenin signalling pathway [43]. This pathway is well‐established in the regulation of proliferation, stemness, and therapy resistance in GB, and its inhibition may contribute to the sensitization of tumour cells to TMZ [44, 45]. In line with this, the combined treatment resulted in a marked downregulation of the Wnt/β‐catenin signalling pathway, as evidenced by decreased expression of β‐catenin and Wnt‐1 proteins in U87 cells. Given the pivotal role of glycogen synthase kinase 3 beta (GSK3β) in regulating β‐catenin stability through its phosphorylation and subsequent degradation [46], we also evaluated both total GSK3β and its phosphorylated form at Ser9 (p‐GSK3β). The combinatory treatment led to a reduction in p‐GSK3β (Ser9)/total GSK3β levels, further supporting the inhibition of the β‐catenin signalling pathway. β‐catenin is known to regulate the transcription of several genes involved in cell stemness, including Nestin, in cancer [47, 48, 49]. The expression of stemness and differentiation markers is significantly altered in GB [50]. In the U87 monolayer model, Nestin, a well‐known marker of neural progenitor cells, was downregulated following combination treatment as well as β3‐tubulin, a marker of neuronal differentiation and glioma aggressiveness [51, 52]. These findings indicate that beyond its cytotoxic effect, HCE may also modulate the Wnt/β‐catenin signalling pathway and the differentiation status of GB cells. In addition to reducing cell viability and stemness features, the combined treatment of HCE and TMZ induced significant mitochondrial damage, which contributed to the activation of the intrinsic apoptotic pathway in GB cells. Mitochondria play a central role in regulating apoptosis, and their dysfunction is often associated with loss of membrane potential, increased ROS production, and activation of pro‐apoptotic signalling cascades [53]. Our data demonstrated that the combined therapy increased the number of Bax‐positive cells, a key pro‐apoptotic member of the Bcl‐2 family. Bax activation and translocation to the mitochondrial outer membrane are critical steps in permeabilization and cytochrome c release, leading to cell death [54]. The TUNEL assay further confirmed the induction of apoptosis, showing a higher number of TUNEL‐positive cells in the combination treatment group compared to each treatment alone. These findings were supported by a clear downregulation of the anti‐apoptotic protein Bcl‐2 in U87 monolayer cells treated with the combination, suggesting that the combined therapy effectively shifts the balance toward a pro‐apoptotic environment, favouring mitochondrial dysfunction and programmed cell death.

GB cells are known to exist in a state of chronic oxidative stress, driven by high metabolic activity, rapid proliferation, and dysfunctional mitochondria [55]. This results in an altered redox homeostasis, characterized by excessive production of ROS and a diminished antioxidant capacity, which collectively contribute to genomic instability, therapy resistance, and tumor aggressiveness [55].

In our study, we observed that the combined treatment with HCE and TMZ significantly altered the redox status of GB cells. Interestingly, the combination therapy led to a reduction in ROS production and ROMO‐1 levels, a mitochondrial protein involved in ROS generation, suggesting a rebalancing of the redox environment. In addition to ROS, lipid peroxidation, a hallmark of oxidative damage, was evaluated through the measurement of MDA levels. The combination of HCE and TMZ significantly reduced MDA content, indicating decreased oxidative damage to cellular membranes. Importantly, this reduction in oxidative stress was accompanied by a notable increase in GSH levels, the main endogenous antioxidant within cells, suggesting that the combination therapy not only reduces ROS generation but also strengthens the antioxidant defense system, contributing to the overall redox balance.

Moreover, we extended our investigation to the nitrosative stress response, which is often elevated in GB and contributes to tumour progression [56, 57], showing that the combination treatment reduced the levels of 3‐NT and nitrite, important markers of nitrosative stress.

To further validate the anti‐tumour efficacy of HCE and TMZ combination, we employed 3D spheroid models, which more accurately mimic the in vivo TME compared to conventional 2D monolayer cultures. Our results demonstrated that the combination of HCE and TMZ induced a significant reduction in spheroid area over time, compared to the control group and the individual treatments. This finding reflects a clear inhibition of tumour‐like growth in the 3D context, reinforcing the synergistic antiproliferative effect observed in monolayer cultures. In parallel, PI‐positive cells were significantly increased in the combination group, particularly in the core region of the spheroid, suggesting a marked induction of cell death throughout the spheroid volume, not limited to the periphery. This indicates that the combined treatment was more effective at penetrating and killing cells in the 3D structure. Interestingly, analysis revealed an increase in p53, a key tumour suppressor involved in the DNA damage response, in spheroids treated with the combination. This upregulation in response to the combined therapy suggests an enhanced activation of apoptotic pathways and cellular stress responses, further supporting the pro‐apoptotic effects induced by HCE and TMZ. Taken together, our data reinforce that HCE exerts measurable anti‐tumoral effects in vitro, including in 3D GB spheroids model. However, several limitations of this study need to be highlighted, including the lack of pharmacokinetic data and the absence of antitumor efficacy evaluation in orthotopic GB models. Future in vivo studies will be required to assess bioavailability and biodistribution. Given that escin is a high–molecular‐weight saponin and therefore unlikely to be able to cross BBB, additional delivery strategies such as nanoparticle‐based formulations or alternative administration routes may be necessary to enable effective CNS targeting.

## 5 Conclusions

In summary, our study demonstrates that the combination of HCE and TMZ exerts a synergistic anti‐tumour effect in GB cells, both in monolayer and 3D spheroid models, through inhibition of the Wnt/β‐catenin signalling pathway and downregulation of stemness and glioma aggressiveness markers. These findings highlight the potential of HCE as an adjuvant to TMZ in GB. However, despite these encouraging in vitro observations, additional validation is required. In vivo studies in tumour‐bearing mice will be essential to define pharmacokinetics and therapeutic activity, especially in an orthotopic context. In parallel, the use of patient‐derived GB cells will be important to confirm that the observed effects are maintained in clinically relevant cellular systems. These steps will be necessary to determine whether the promising biological activity of HCE observed in this study can be substantiated beyond the in vitro setting.

## Author Contributions

Conceptualization: I.P. Methodology: D.M., N.P., F.D.L., and A.C. Data curation: S.A.S., and A.A. Writing – original draft preparation: S.A.S. and A.A. Writing – review and editing: I.P. Supervision: M.C. and E.E. All authors have read and agreed to the published version of the manuscript.

## Funding

The authors have nothing to report.

## Ethics Statement

The authors have nothing to report.

## Conflicts of Interest

The authors declare no conflicts of interest.

## Supporting information


**Figure S1:** Assessment of HCE cytotoxicity in association with TMZ in A172 cells. Cell viability was assessed by MTT assay after 24 h and 48 h of treatment with increasing concentrations of HCE (62.5, 125, 250, 500, and 800 μg/mL), alone or in combination with TMZ (100 μM) (A–B). Data are representative of three independent experiments.
**Figure S2:** HCE and TMZ combination impairs migration and clonogenic ability of A172 cells. Wound healing assay (A–E) and colony formation assay (F–J, F1‐I1) were performed in A172 cells treated with HCE (500 μg/mL) and TMZ (100 μM), alone or in combination, for 48 h. Data are representative of three independent experiments. Images were captured at 2× and 10× magnifications. One‐Way ANOVA. (E) ***p < 0.001 versus Control; ##*p* < 0.01 versus TMZ. (J) **p < 0.01 versus Control; ***p < 0.001 versus Control.
**Figure S3:** HCE and TMZ combination increases apoptosis in A172 cells. Western blot analysis was performed to evaluate the expression of Bax and p53 in A172 cell line (A, B; A1, B1). Data are representative of three independent experiments. (A1) ***p < 0.001 versus Control; ##*p* < 0.01 versus TMZ. (B1) *p < 0.05 versus Control; ***p < 0.001 versus Control; #*p* < 0.05 versus TMZ.

**Table S1:** Quali‐quantitative composition of *Aesculus hippocastanum* dry extract per 100 g, and expressed as % w/w.

## Data Availability

The data supporting the findings of this study are available from the corresponding author(s) upon reasonable request.
