# Supplementary figures and images for: Modulation of Wnt/β‐Catenin Pathway by Aesculus hippocastanum Extract Enhances Temozolomide Sensitivity in Glioblastoma Cells
Source: J Cell Mol Med. 2026 Feb 16;30(4):e70979. doi: 10.1111/jcmm.70979 (PMC12907738; doi:10.1111/jcmm.70979)

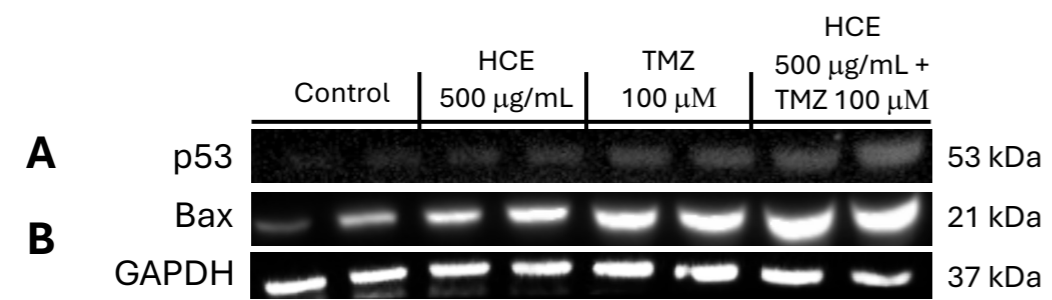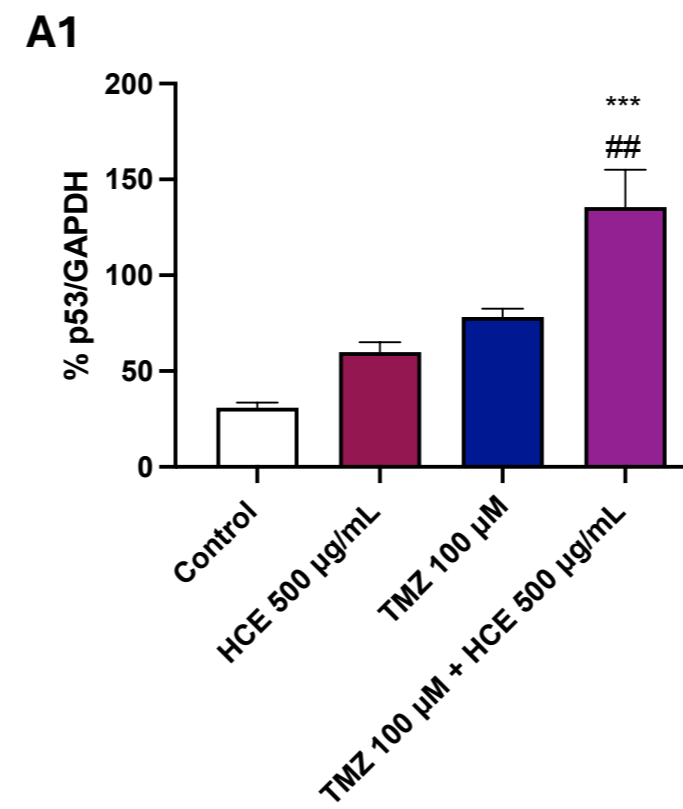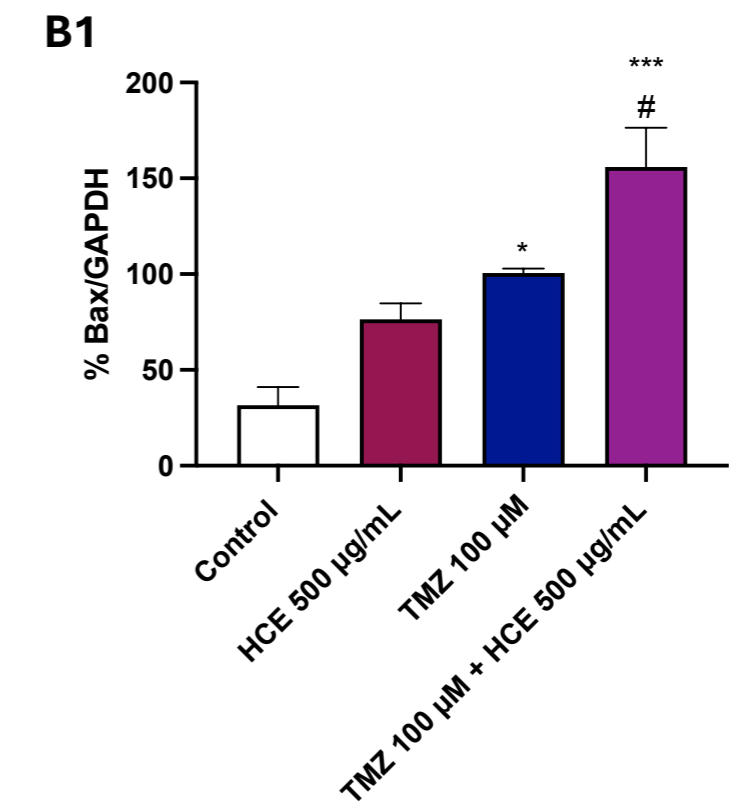

Supplementary Figure 3

Supplement: Supplementary file 1 — Figure S1: Assessment of HCE cytotoxicity in association with TMZ in A172 cells. Cell viability was assessed by MTT assay after 24 h and 48 h of treatment with increasing concentrations of HCE (62.5, 125, 250, 500, and 800 μg/mL), alone or in combination with TMZ (100 μM) (A–B). Data are representative of three independent experiments. Figure S2: HCE and TMZ combination impairs migration and clonogenic ability of A172 cells. Wound healing assay (A–E) and colony formation assay (F–J, F1‐I1) were performed in A172 cells treated with HCE (500 μg/mL) and TMZ (100 μM), alone or in combination, for 48 h. Data are representative of three independent experiments. Images were captured at 2× and 10× magnifications. One‐Way ANOVA. (E) ***p < 0.001 versus Control; ##p < 0.01 versus TMZ. (J) **p < 0.01 versus Control; ***p < 0.001 versus Control. Figure S3: HCE and TMZ combination increases apoptosis in A172 cells. Western blot analysis was performed to evaluate the expression of Bax and p53 in A172 cell line (A, B; A1, B1). Data are representative of three independent experiments. (A1) ***p < 0.001 versus Control; ##p < 0.01 versus TMZ. (B1) *p < 0.05 versus Control; ***p < 0.001 versus Control; #p < 0.05 versus TMZ. [file JCMM-30-e70979-s001.zip › SF 3.pdf]

**A**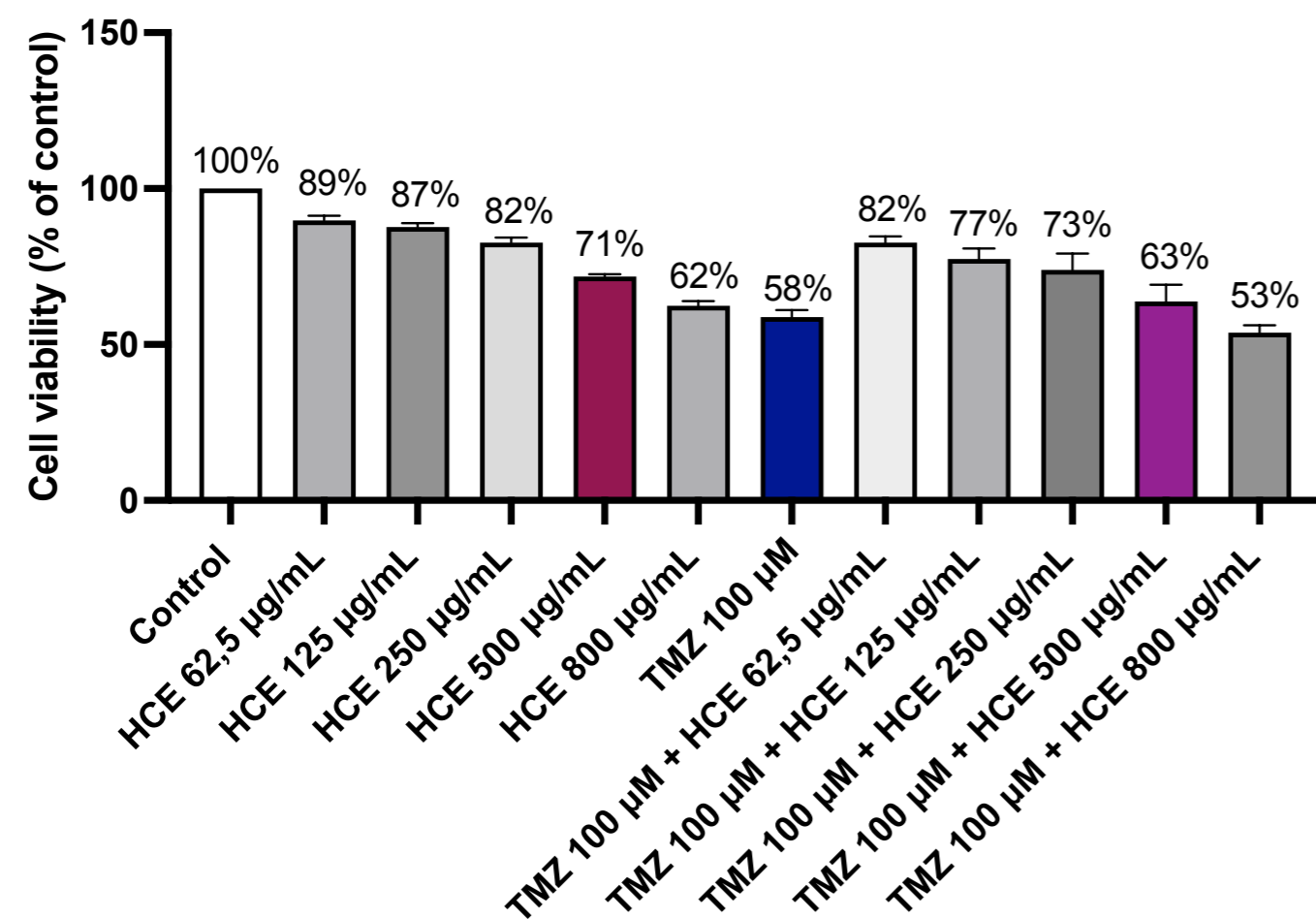**B**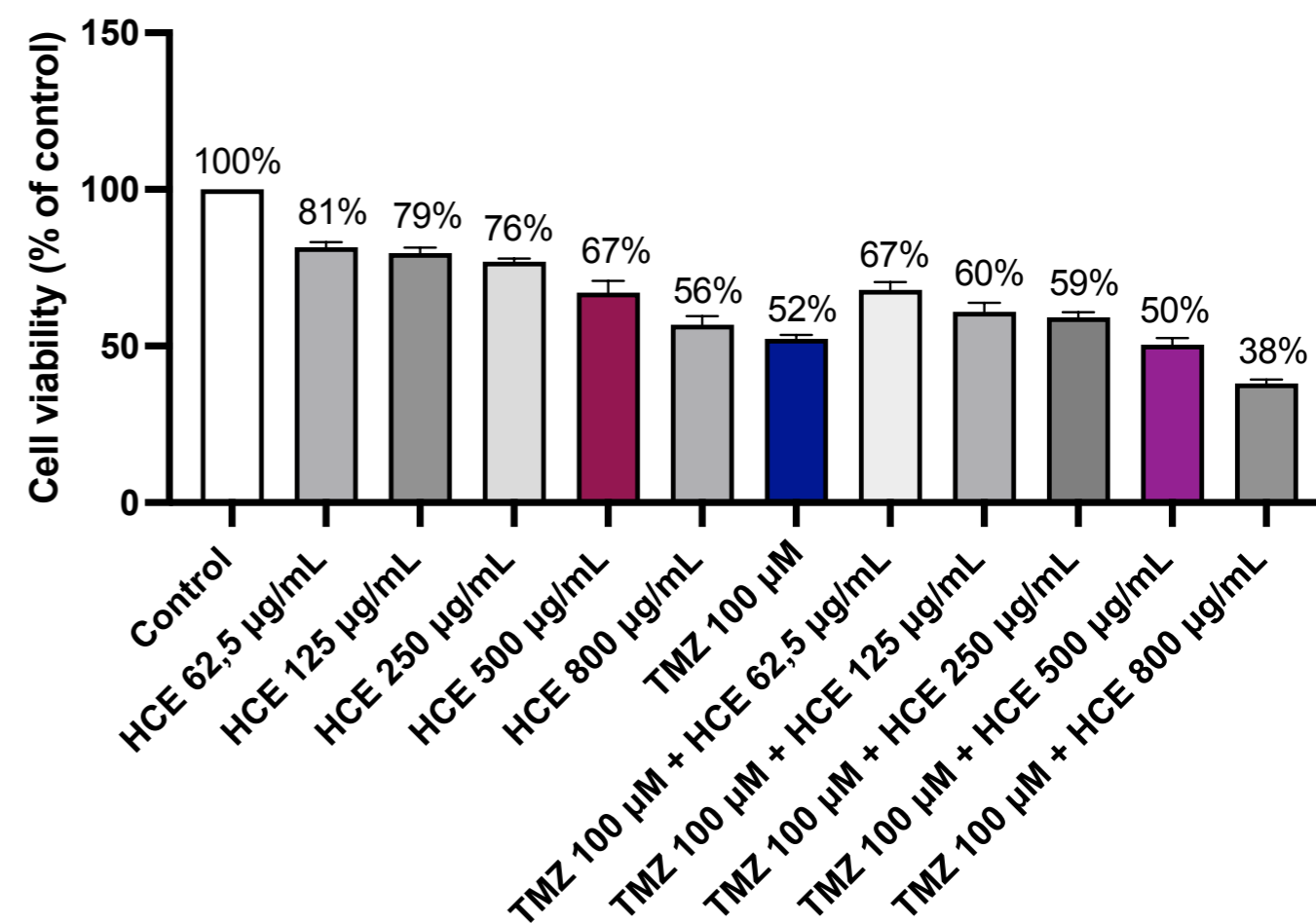

Supplement: Supplementary file 1 — Figure S1: Assessment of HCE cytotoxicity in association with TMZ in A172 cells. Cell viability was assessed by MTT assay after 24 h and 48 h of treatment with increasing concentrations of HCE (62.5, 125, 250, 500, and 800 μg/mL), alone or in combination with TMZ (100 μM) (A–B). Data are representative of three independent experiments. Figure S2: HCE and TMZ combination impairs migration and clonogenic ability of A172 cells. Wound healing assay (A–E) and colony formation assay (F–J, F1‐I1) were performed in A172 cells treated with HCE (500 μg/mL) and TMZ (100 μM), alone or in combination, for 48 h. Data are representative of three independent experiments. Images were captured at 2× and 10× magnifications. One‐Way ANOVA. (E) ***p < 0.001 versus Control; ##p < 0.01 versus TMZ. (J) **p < 0.01 versus Control; ***p < 0.001 versus Control. Figure S3: HCE and TMZ combination increases apoptosis in A172 cells. Western blot analysis was performed to evaluate the expression of Bax and p53 in A172 cell line (A, B; A1, B1). Data are representative of three independent experiments. (A1) ***p < 0.001 versus Control; ##p < 0.01 versus TMZ. (B1) *p < 0.05 versus Control; ***p < 0.001 versus Control; #p < 0.05 versus TMZ. [file JCMM-30-e70979-s001.zip › SF 1.pdf]

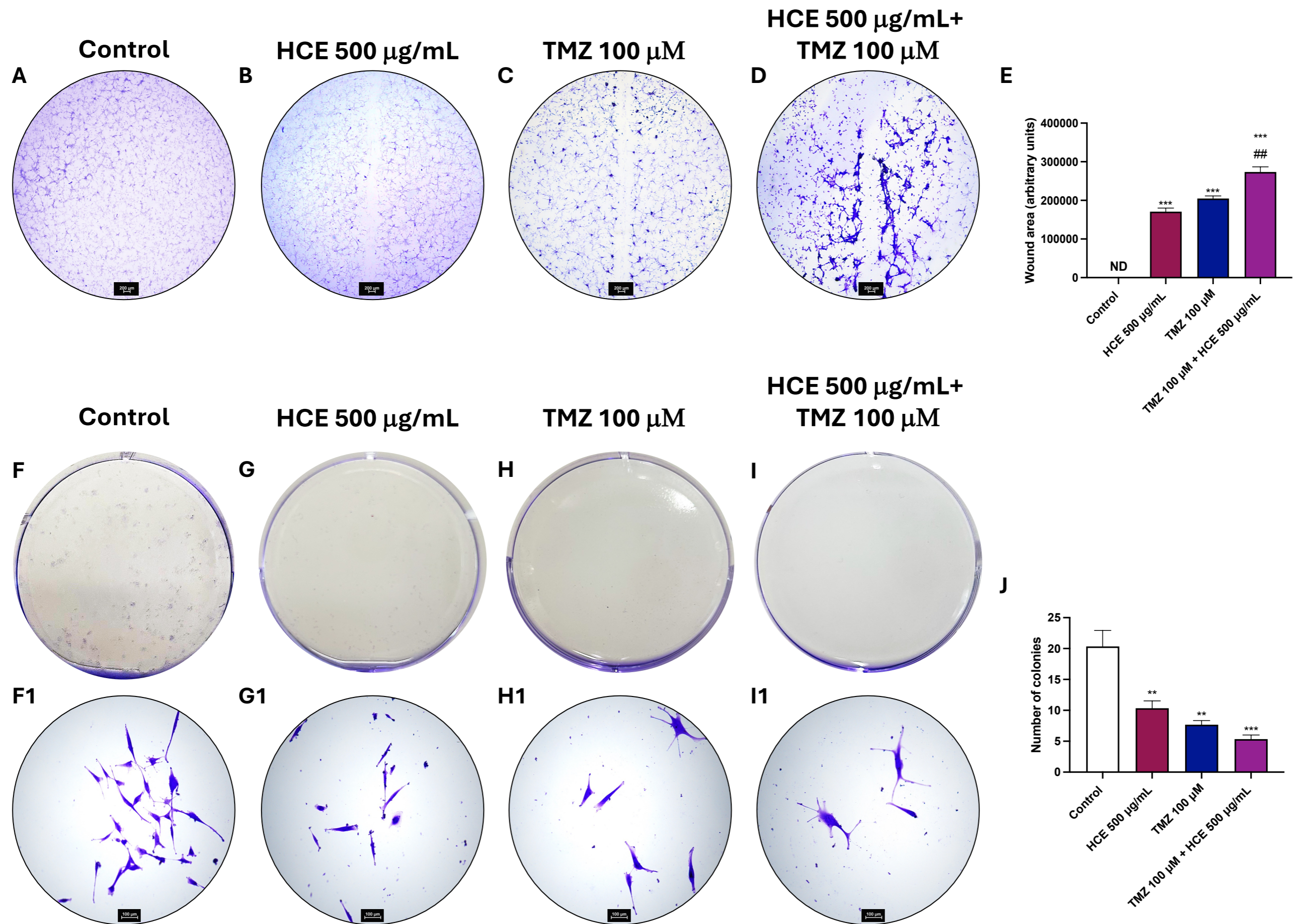

**Supplementary Figure 2**

Supplement: Supplementary file 1 — Figure S1: Assessment of HCE cytotoxicity in association with TMZ in A172 cells. Cell viability was assessed by MTT assay after 24 h and 48 h of treatment with increasing concentrations of HCE (62.5, 125, 250, 500, and 800 μg/mL), alone or in combination with TMZ (100 μM) (A–B). Data are representative of three independent experiments. Figure S2: HCE and TMZ combination impairs migration and clonogenic ability of A172 cells. Wound healing assay (A–E) and colony formation assay (F–J, F1‐I1) were performed in A172 cells treated with HCE (500 μg/mL) and TMZ (100 μM), alone or in combination, for 48 h. Data are representative of three independent experiments. Images were captured at 2× and 10× magnifications. One‐Way ANOVA. (E) ***p < 0.001 versus Control; ##p < 0.01 versus TMZ. (J) **p < 0.01 versus Control; ***p < 0.001 versus Control. Figure S3: HCE and TMZ combination increases apoptosis in A172 cells. Western blot analysis was performed to evaluate the expression of Bax and p53 in A172 cell line (A, B; A1, B1). Data are representative of three independent experiments. (A1) ***p < 0.001 versus Control; ##p < 0.01 versus TMZ. (B1) *p < 0.05 versus Control; ***p < 0.001 versus Control; #p < 0.05 versus TMZ. [file JCMM-30-e70979-s001.zip › SF 2.pdf]
