# Supplementary material for: Modulation of Wnt/β‐Catenin Pathway by Aesculus hippocastanum Extract Enhances Temozolomide Sensitivity in Glioblastoma Cells
Source: J Cell Mol Med. 2026 Feb 16;30(4):e70979. doi: 10.1111/jcmm.70979 (PMC12907738; doi:10.1111/jcmm.70979)
Supplement: Supplementary file 2 — Table S1: Quali‐quantitative composition of Aesculus hippocastanum dry extract per 100 g, and expressed as % w/w. [file JCMM-30-e70979-s002.pdf]

**Components**	**% *w/w***
Escin	19.55 %
Total flavonoids fraction	2.84 %
Tannins	3.06 %
Total Coumarins fraction	0.91%
Polysaccharides and soluble sugars	14.53 %
Inert plant material	54.25 %
Residual moisture	4.86 %

Supplementary Table 1. Quali-quantitative composition of *Aesculus hippocastanum* dry extract per 100 g, and expressed as % *w/w*,
